# Anthracycline Drugs on Modified Surface of Quercetin-Loaded Polymer Nanoparticles: A Dual Drug Delivery Model for Cancer Treatment

**DOI:** 10.1371/journal.pone.0155710

**Published:** 2016-05-19

**Authors:** Chabita Saha, Agrima Kaushik, Asmita Das, Sandip Pal, Debashis Majumder

**Affiliations:** Department of Biotechnology, Maulana Abul Kalam Azad University of Technology, BF-142, Salt Lake, Sector-I, Kolkata 700 064, West Bengal, India; University of Quebec at Trois-Rivieres, CANADA

## Abstract

Polymer nanoparticles are vehicles used for delivery of hydrophobic anti-cancer drugs, like doxorubicin, paclitaxel or chemopreventors like quercetin (Q). The present study deals with the synthesis and characterisation of nano formulations (NFs) from Q loaded PLGA (poly lactic-co-glycolic acid) nano particles (NPs) by surface modification. The surface of Q-loaded (NPs) is modified by coating with biopolymers like bovine serum albumin (BSA) or histones (His). Conventional chemotherapeutic drugs adriamycin (ADR) and mitoxantrone (MTX) are bound to BSA and His respectively before being coated on Q-loaded NPs to nano formulate NF1 and NF2 respectively. The sizes of these NFs are in the range 400–500 nm as ascertained by SEM and DLS measurements. Encapsulation of Q in polymer NPs is confirmed from shifts in FT-IR, TGA and DSC traces of Q-loaded NPs compared to native PLGA and Q. Surface modification in NFs is evidenced by three distinct regions in their TEM images; the core, polymer capsule and the coated surface. Negative zeta potential of Q-loaded NPs shifted to positive potential on surface modification in NF1 and NF2. *In vitro* release of Q from the NFs lasted up to twenty days with an early burst release. NF2 is better formulation than NF1 as loading of MTX is 85% compared to 23% loading of ADR. Such NFs are expected to overcome multi-drug resistance (MDR) by reaching and treating the target cancerous cells by virtue of size, charge and retention.

## Introduction

**Fig 1:**
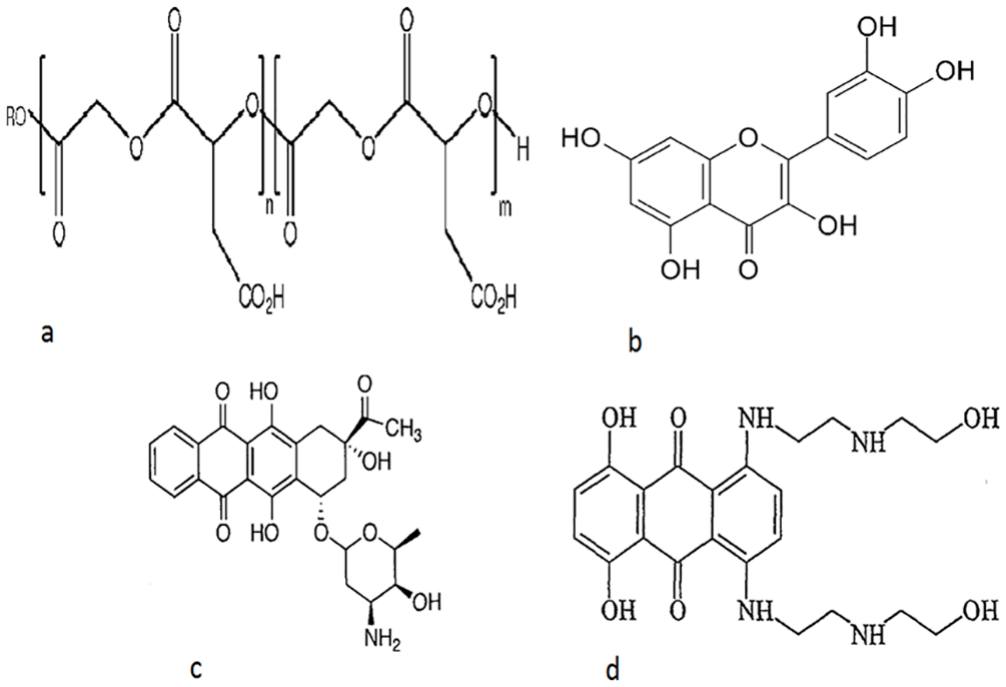
Chemical structures. (a) PLGA, (b) Quercetin, (c) Adriamycin and (d) Mitoxantrone.

Most anti-cancer drugs have limitations in clinical administration due to their poor solubility, some physicochemical and pharmaceutical properties. They require the use of adjuvant, which often cause serious side effects when administered intravenously. Considerable changes in concentration of this adjuvant are recorded in the blood plasma. These limitations are overcome through nano formulation using biodegradable polymers and bioadhesive materials to encapsulate anticancer drug and render them suitable for oral administration. Many biopolymers like chitosan, gelatine, proteins and lipids are used for drug encapsulation. In the present work we have used Poly (lactic-co-glycolic acid) PLGA (Fig 1a) as a polymer for drug encapsulation. PLGA is approved by the US FDA for drug delivery because of its biodegradability, efficacy of encapsulating hydrophobic drugs and sustained release of the drug at the target site. Encapsulation shields the drugs from chemical degradation and non specific binding. The size of these nanoparticles enables them to penetrate specific cancerous cells via receptors and or other pathways which are over expressed by target cells. Various formulations of drug loaded polymer nanoparticles and their efficacy in cancer treatment is reported [1–5].

Our interest is to deliver more than one drug by surface modification of drug encapsulated PLGA NPs. This delivery model is designed to overcome multi-drug resistance (MDR) which is major impediment in cancer treatment. In this model the hydrophobic drug is encapsulated in the core of the NPs and its surface is modified to accommodate a hydrophilic drug. The surface is modified by coating a biopolymer to which the hydrophilic drug is bound. Biopolymers like BSA or His can have dual role; one is to carry the drug and other is to shield the nanoparticles from body immune system, reticulo endothelial system (RES) and cellular degradation. Dietary polyphenols are bestowed with chemopreventive properties and are ubiquitously found in fruits and vegetables. Quercetin (Q) [6], a hydrophobic phenolic antioxidant is encapsulated in PLGA NPs. It has a chemical structure (Fig 1b) that counteracts the damaging effects of oxidation caused by ROS or free radicals in living cells of our body. The suppression of carcinogenesis is suggested to be due to its radical scavenging activity. Q is also known to have chemopreventive properties along with ability to reverse the MDR pathways [7, 8]. Q is also reported to inhibit CYP450, COX protein and tyrosine kinase family of enzymes leading to modulation of signal transduction and apoptosis [9]. Adriamycin (ADR) and mitoxantrone (MTX) (Fig 1c and 1d respectively) are well known chemotherapeutic drugs of the anthracyclines group. They are used as the hydrophilic drugs which act by DNA intercalation leading to apoptosis [10–12]. When chemotherapeutic drugs like ADR/MTX act on cancerous cells some normal cells in the near vicinity are also sacrificed; Q can help in reducing the damage as an antioxidant. In cancerous cells Q can reverse MDR and render them favourable for the action of ADR and MTX. The drugs when delivered in combination can either work synergistically or as adjuvant. In the present work, the synthesis of Q-loaded polymer NPs carrying second drug on modified surface and their physicochemical analysis is reported. Such formulations can be used as alternative anti-cancer drug delivery system to over come MDR.

## Materials and Methods

PLGA (LA:GA-50:50) of molecular weight 30,000–60,000, quercetin, histone, mitoxantrone, adriamycin, and sodium azide were obtained from sigma. PVA (poly-vinyl alcohol) was obtained from Aldrich and BSA from Merck. Acetone used was of analytical grade from Merck. Milli Q water and phosphate buffer from sigma was used where ever necessary.

### Preparation of drug loaded NPs

**Fig 2:**
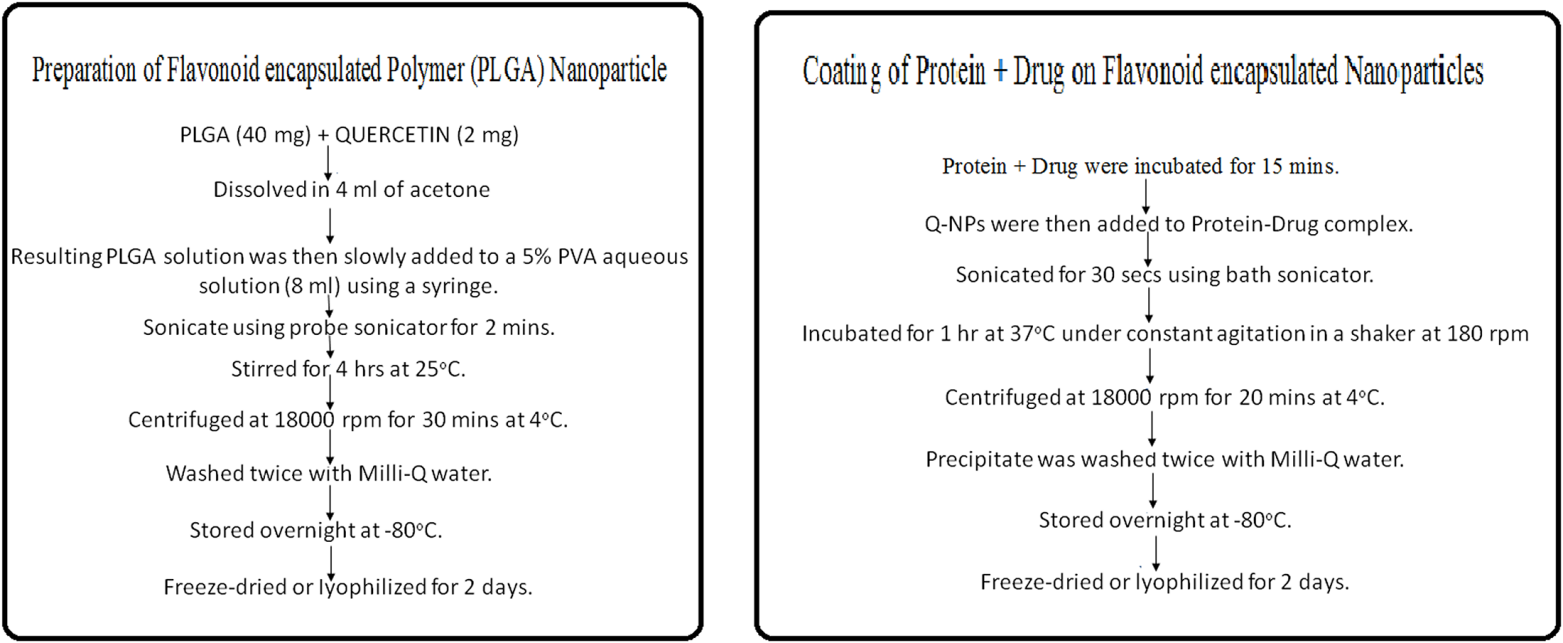
Single Emulsion Solvent Evaporation Technique. Schematic representation of the synthesis and surface modification of PLGA NPs.

PLGA nanoparticles were prepared by “*Single Emulsion Solvent Evaporation Technique*” [13] as summarised in Fig 2. PLGA (40 mg) and Q (2 mg) was dissolved in 4 ml of acetone. The resulting PLGA solution was then slowly added to a 5% PVA aqueous solution (8 ml) using a syringe. The solution was then sonicated using a probe sonicator (Hielscher UP100H, Germany) over ice bath for 2 min. The emulsion was stirred for 4 hrs at 25°C on a magnetic stir plate to allow evaporation of organic solvent. The formed nanoparticles were recovered by ultracentrifugation at 18000 rpm for 30 mins at 4°C and washed twice with Milli-Q water to remove unbound or excess PVA and free Q. The washed formulations were stored overnight at -80°C in a freezer and then freeze-dried or lyophilized (Gemini^BV^ Heto Maxi Dry Lyo) for 2 days to get the powdered form of NPs.

### Surface modification of NPs

BSA binds to ADR with binding constant 7.8 x 10^3^ M^-1^ [14] and has been coated on supramagnet iron oxide nanoparticles to bind to drugs[15]. From the binding constant the ratio of their binding concentration was determined. Accordingly ADR (2.2 × 10^−4^ M) was incubated with BSA (1 mg/ml) for 15 mins. Q-NPs were then added to BSA-ADR complex and the resulting mixture was sonicated for 30 s using bath sonicator. The resulting mixture was then incubated for 1 hr at 37°C under constant agitation in a shaker at 180 rpm to facilitate the adsorption process [3,16,17]. The BSA-ADR coated and Q-loaded NPs were then recovered by ultracentrifugation for 20 mins at 4°C. Washed twice with Milli-Q water to remove unbound BSA and free ADR. The washed formulations were stored overnight at -80°C in a freezer and then freeze-dried or lyophilized for 2 days to get the powdered form of NPs. Similarly the second formulation was prepared using histone and MTX in 5:1 ratio calculated on the basis of their binding constants.

### Particle size analysis and zeta potential measurements

Dynamic laser scattering (DLS) was used to measure the hydrodynamic diameter (nm), and Laser Doppler Anemometry (LDA) was used to determine zeta potential (mV). The DLS and LDA analyses were performed using Zetasizer Nano ZS (Malvern Instruments, Malvern, UK). To determine the particle size and zeta potential, a dilute suspension of void NPs, Q-loaded NPs, NF1 and NF2 (100 μg/ml) each was prepared in double distilled water, sonicated on an ice-bath for 30 s and subjected to particle size and zeta potential measurement separately. All measurements were performed in duplicates.

### Determination of drug entrapment efficiency

The entrapment efficiency (E%) of Q-loaded in PLGA nanoparticles was determined in the following method: the nanoparticles were separated from the free drug by centrifugation and the amount of free drug in the supernatant was measured using spectrophotometer. The E% was calculated by the following equation:

$$E\%=\;({\left[Drug\right]}_{total}-{\left[Drug\right]}_{free})\;/\;{\left[Drug\right]}_{total}\times \;100,$$

where drug is Q.

### Determination of drug loading efficiency

Loading efficiency was calculated by *L*% = ([*Drug*]_*total*_ / [*NP*]_*total*_) × 100, where drug is Q.

### Fourier transformed infrared spectroscopy

The Fourier transformed infrared spectroscopic (FT-IR) analysis was conducted to verify the presence of various chemical functional groups in PLGA, Q, Q-loaded PLGA NPs and surface modified NFs. The FT-IR spectra were recorded with Jasco Fourier Transform Infrared Spectrometer. Dry solid samples (1% by weight) were finely crushed and mixed with potassium bromide and pressed to make a pallet. Scans were recorded for each sample at a spectral range from 4000–400 cm^-1^.

### Differential scanning calorimetry

Measurements of the thermal behaviour of pure Q, PLGA void NPs and (NFs) were performed with Differential Scanning Calorimeter (Pyris Diamond DSC, Perkin Elmer). The samples were loaded onto standard aluminium pans and were scanned in a range from of 0°C−350°C with a scan rate of 10°C/min.

### Thermogravimetric analysis

Thermogravimetric Analysis (TGA) measures weight changes in a material as a function of temperature (or time) under a controlled atmosphere. Thermogravitometric profile of void PLGA NPs, free Q and Q-loaded NPs were recorded on TGA, TA instrument Q 600 SDT simultaneous DSC TGA.

### In vitro release kinetics study

*In vitro* release of Q from NPs was carried out by dissolving 2 mg of NPs in 1 ml of PBS (0.01 M, pH 7.4) containing 0.1% v/v of NaN_3_ (to maintain a sink condition). The NP suspension was equally divided in two tubes containing 1 ml each (as the experiment was performed in duplicate) and kept in a shaker at 37°C at 150 rpm. At particular time intervals like (1 day, 2 day up to 20 days) these tubes were taken out from shaker and centrifuged at 13,800 rpm, 4°C for 10 min. To the pellet obtained after centrifugation, 1 ml of fresh PBS/ NaN_3_ solution was added to the shaker for the next readings. The collected supernatant was lyophilized and dissolved in 1 ml of DMSO/acetone. The solution was centrifuged at 13,800 rpm for 10 min at 25°C to collect the drug in the supernatant. The amount of Q in the sample was measured fluorimetrically.

### Scanning electron microscope (SEM) studies

The surface morphology of NPs was characterized by SEM (Zeiss Evo-MA 10) operating at an accelerating voltage of 10–30 kV. Few drops of void, Q-loaded NPs, NF1 and NF2 water suspensions (100 μg/ml) were separately dried on small glass pieces and sputtered with gold to make them conductive and placed on a copper stub prior to the acquisition of SEM images.

### Transmission electron microscope (TEM) studies

The internal structure of NPs was determined by TEM (Jeol Jem 2100 HR with EELS). One drop of void, Q-loaded, BSA-ADR NF and His-MTX NF water suspensions (100 μg/ml) placed over a carbon coated copper TEM grid (150 mesh, Ted Pella Inc., Redding, CA), and allowed to dry. The images were visualized at an accelerating voltage of 120 kV under the transmission electron microscope.

## Results

### SEM-TEM analysis

**Fig 3:**
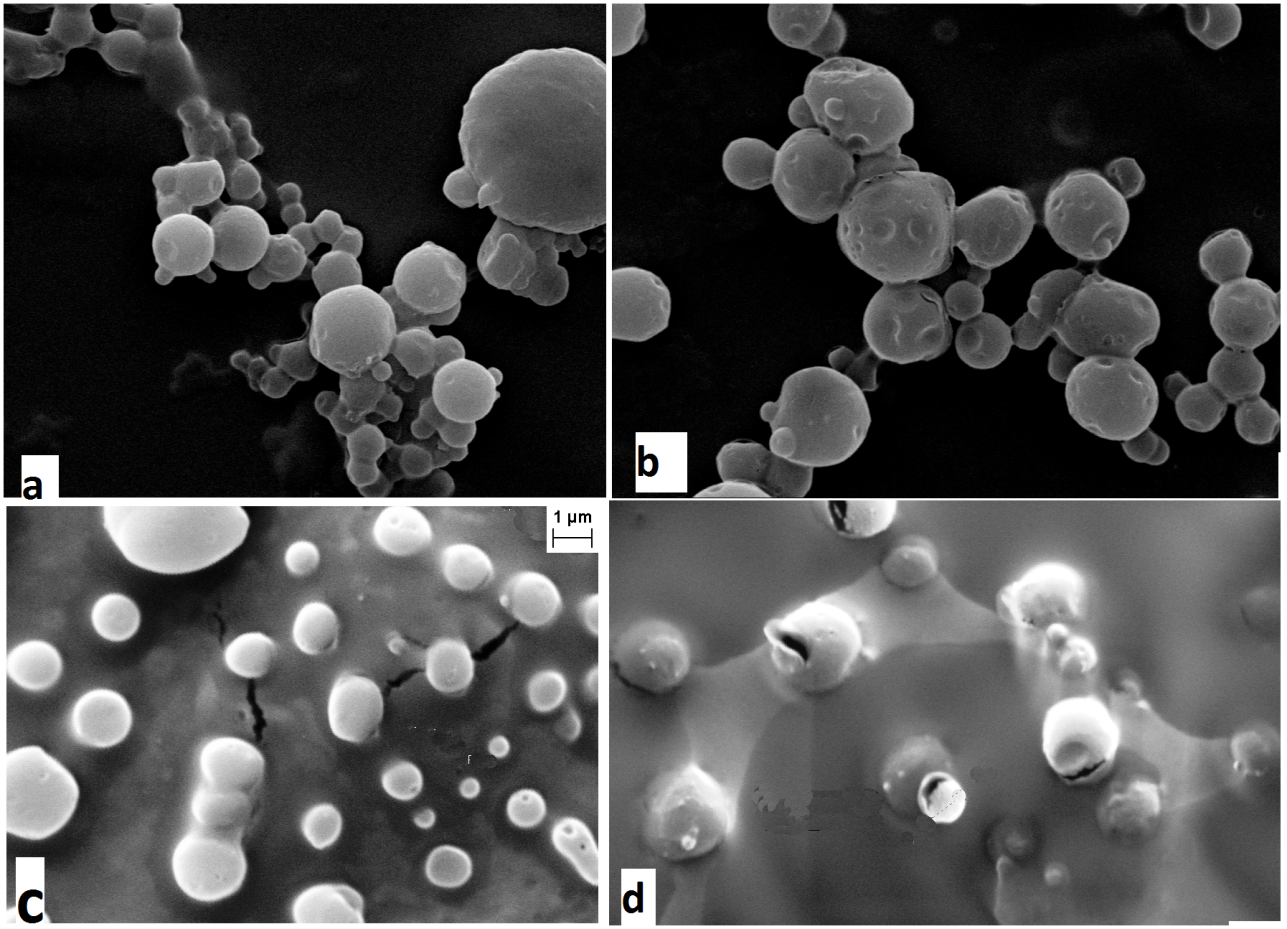
SEM images of NPs and NFs. Scanning electron microscope micrographs of (a) void-PLGA NPs; (b) Q-loaded NPs; (c) NF1 and (d) NF2.

**Fig 4:**
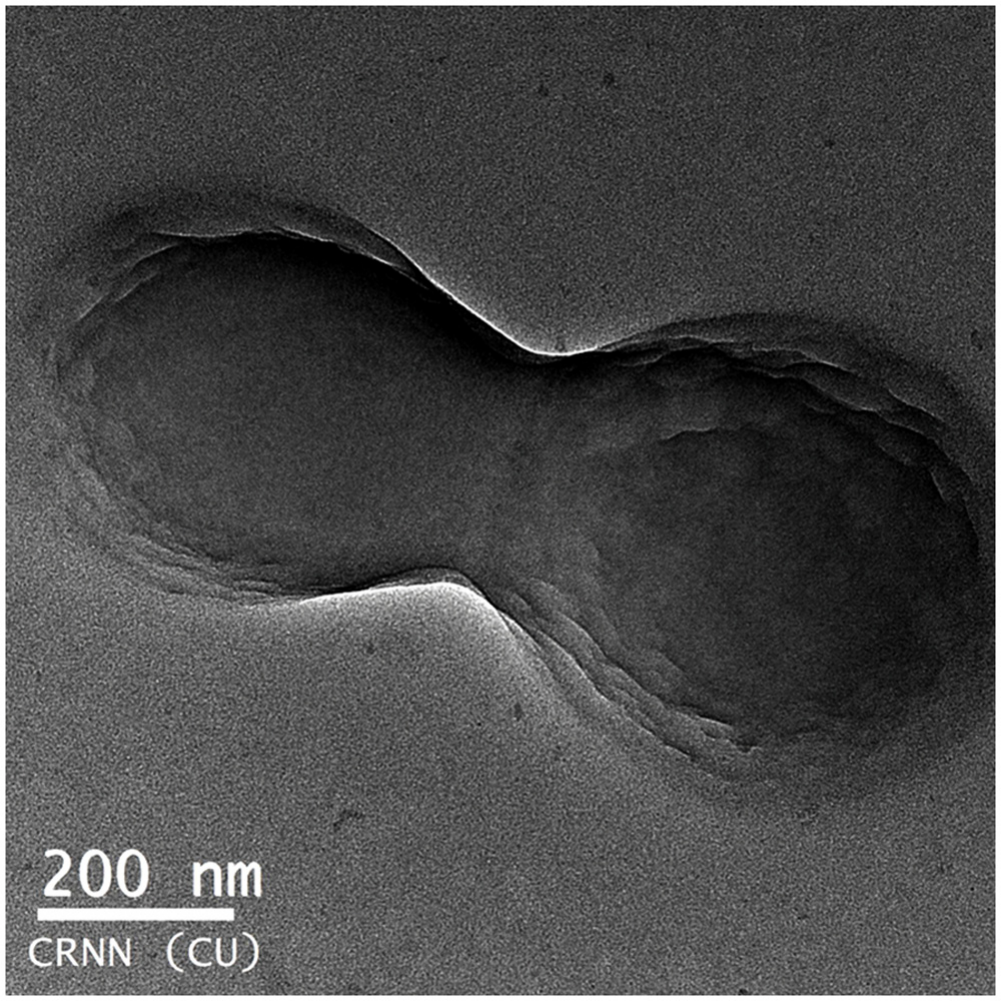
TEM image of NF2. Transmission electron microscope image of NF2 illustrating the three layers of; encapsulated Q, polymer capsule and surface modification.

SEM images of the void NPs and Q-loaded NPs as illustrated in Fig 3a and 3b confirm their formation by the standard solvent evaporation method used. The exterior surface of void is smoother compared to loaded NPs, suggesting encapsulation of the drug. The SEM images of NF1 and NF2 are represented in Fig 3c and 3d respectively. From the figures it is revealed that the sizes of the NFs are greater than the Q-loaded NPs evidencing successful surface modification. The pealing of layer from the surface also confirms the surface coating in NF1 and NF2. TEM images (Fig 4) of the surface modified NF2 show three distinct layers; one is of the drug in the core of the NPs, second is the polymer encapsulation and third is the coating on the surface.

### Size determination and zeta potential measurement

**Fig 5:**
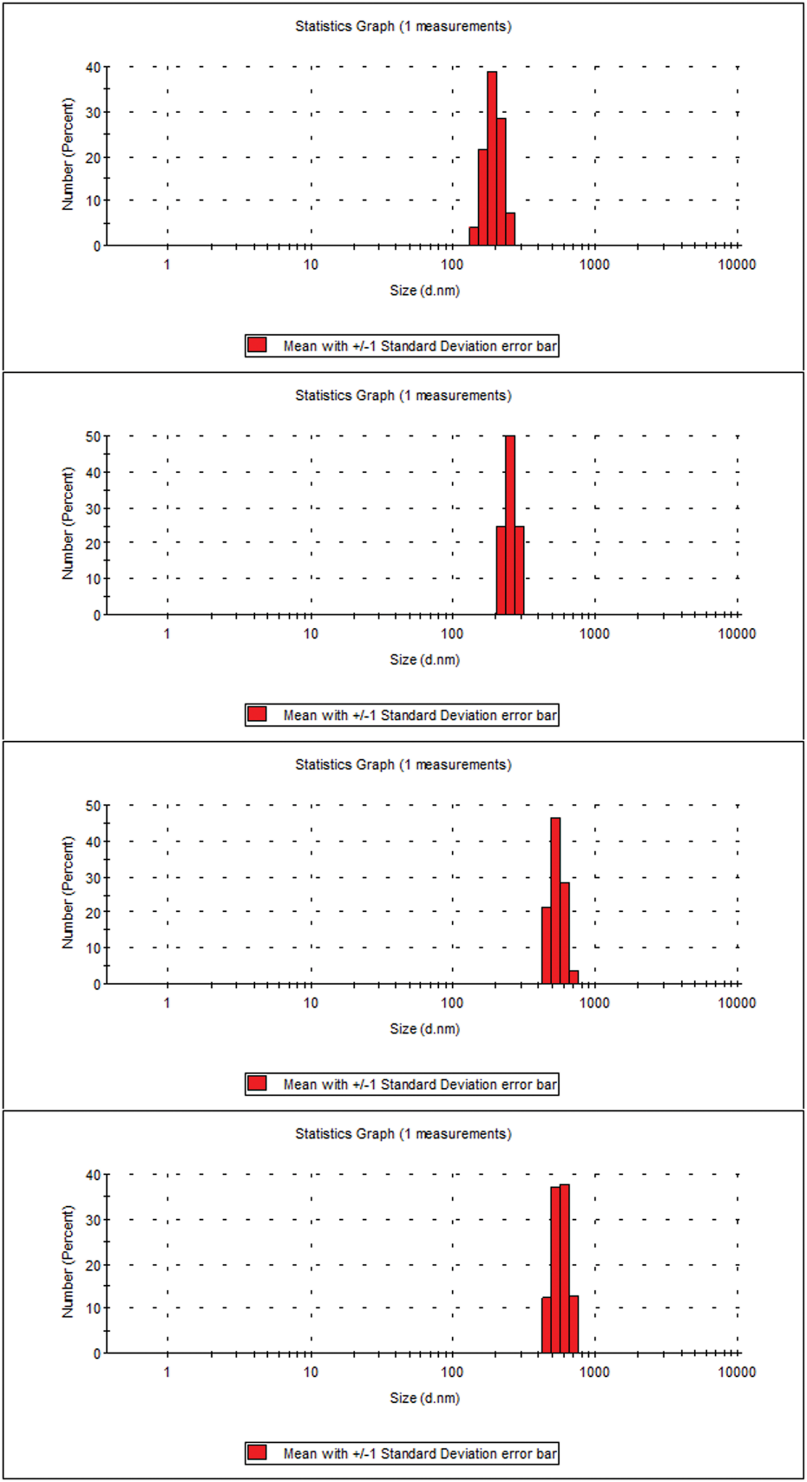
Size distribution of NPs and NFs. Size in nm (a) void-PLGA NPs (b) Q-loaded NPs (c) NF1 and (d) NF2.

Zeta potentials were measured using dynamic light scattering and the values recorded are -2.0 mv, -10.0 mv, +3.0 mv and +8.0 mv respectively for void NPs, Q-loaded NPs, NF1 and NF2 respectively. The shift from negative zeta potential of Q-loaded to positive potential in NF1 and even higher positive shift in NF2 also support successful coating. The measured sizes are about 180 nm for void and 250 nm for Q-loaded NPs. For NF1 and NF2 the sizes are between 400 to 500 nm. The DLS out put of size population for NPs and NFs are reproduced in Fig 5.

### FT-IR analysis

**Fig 6:**
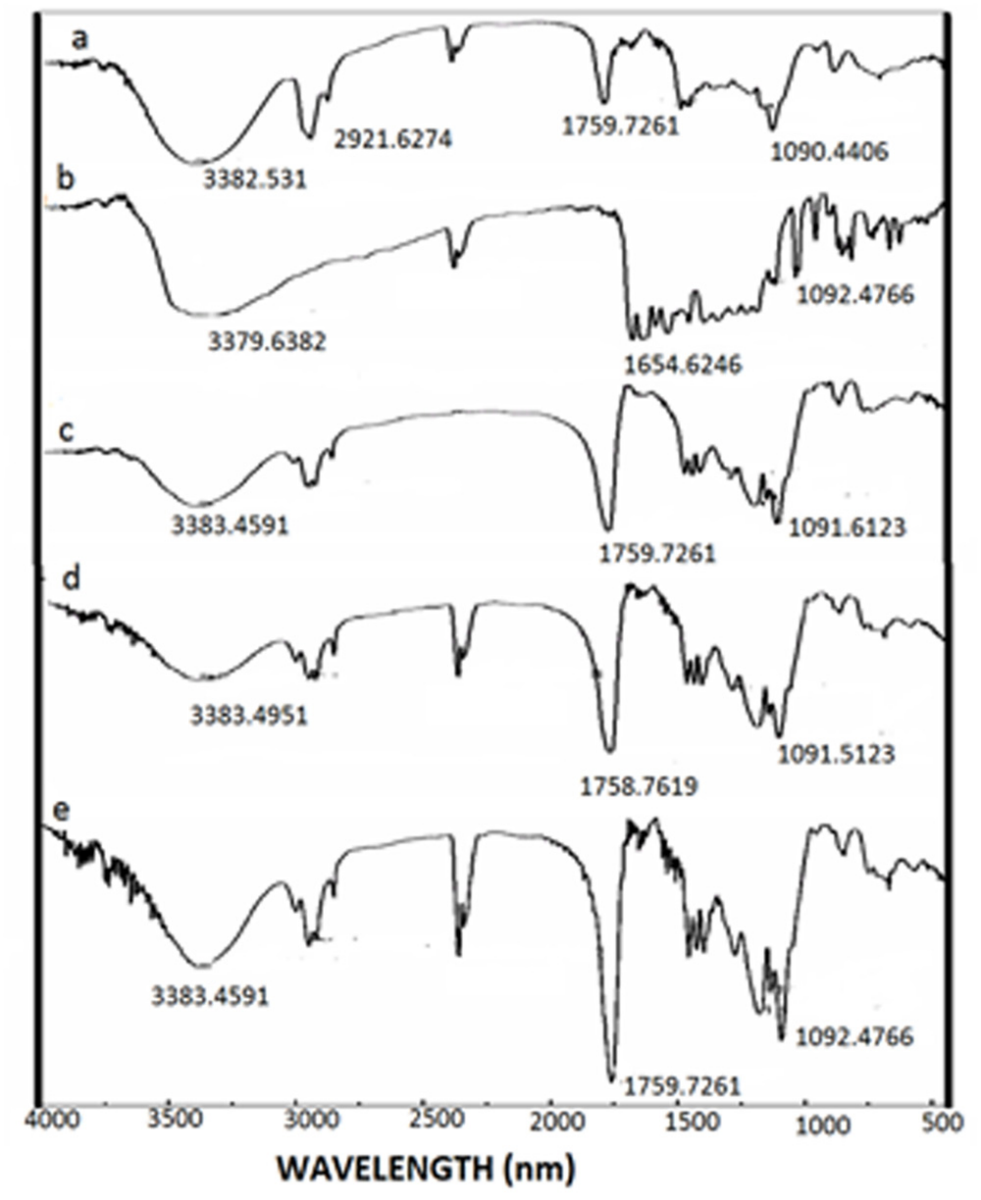
Fourier transformed infrared spectra in the region of 4000–400 cm^-1^. FTIR spectra of (a) pure—Q (b) BSA (c) PLGA (d) NF1 and (e) NF2.

The FT-IR spectra of pure Q, BSA and PLGA are illustrated in Fig 6a, 6b and 6c. The spectra show characteristic peaks of functional groups like OH, CH, C-O and C = O bands in accordance with the reported spectra of Q [18, 19], BSA [20, 21] and PLGA [22]. The FTIR spectra of the surface modified NF1 and NF2 (Fig 6d and 6e respectively) also retained the characteristic bands of PLGA and Q with slight shifts. New bands in the spectra are assigned to the amide from the protein coating and OH contribution from the drugs. Bands in around 3383 cm^-1^ are assigned to OH stretch which is intrinsic of PLGA, Q and BSA. In Fig 6e and 6d bands in the region 3062 cm^-1^ is characteristic of the amide-A of the proteins which is more prominent for NF1 and NF2 which are coated with proteins. The amide I and II bands at 1652 and 1531 cm^-1^ respectively [20] of the proteins are integrated with the PLGA peak at 1758 cm^-1^ and so its intensity is high for NF1 and NF2. Bands between 3000–2850 cm are signature bands of CH stretching common to all the NPs and NFs. The strong bands at 1320–1000 and 1760–1690 cm^-1^ identified in all the NPs are assigned to C-O and C = O bonds present in the carboxyl groups of PLGA.

### TGA and DSC analysis

**Fig 7:**
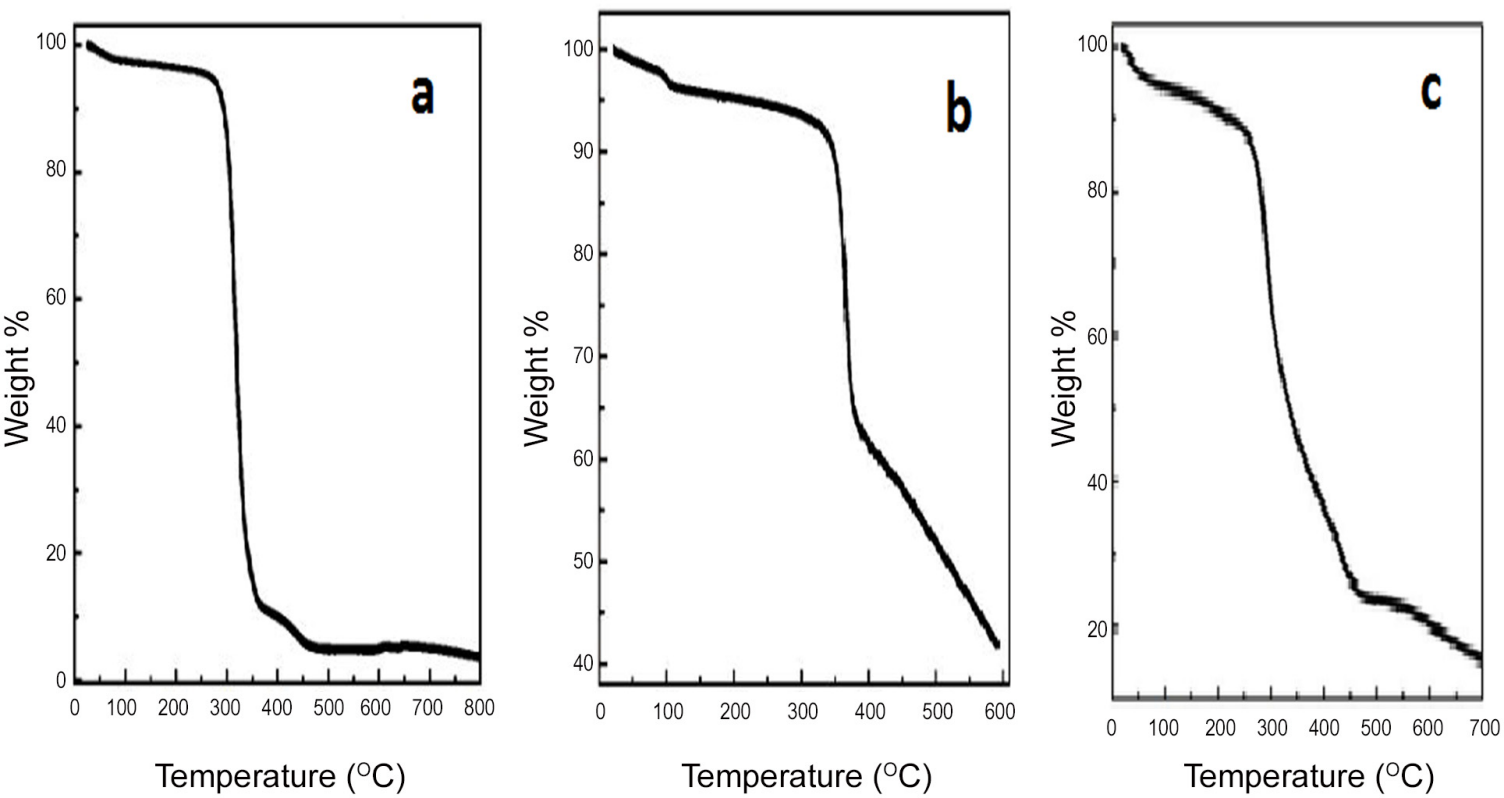
Thermo gravimetric profiles. TGA melting curves of (a) void-PLGA NPs (b) free Q and (c) Q-loaded NPs.

**Fig 8:**
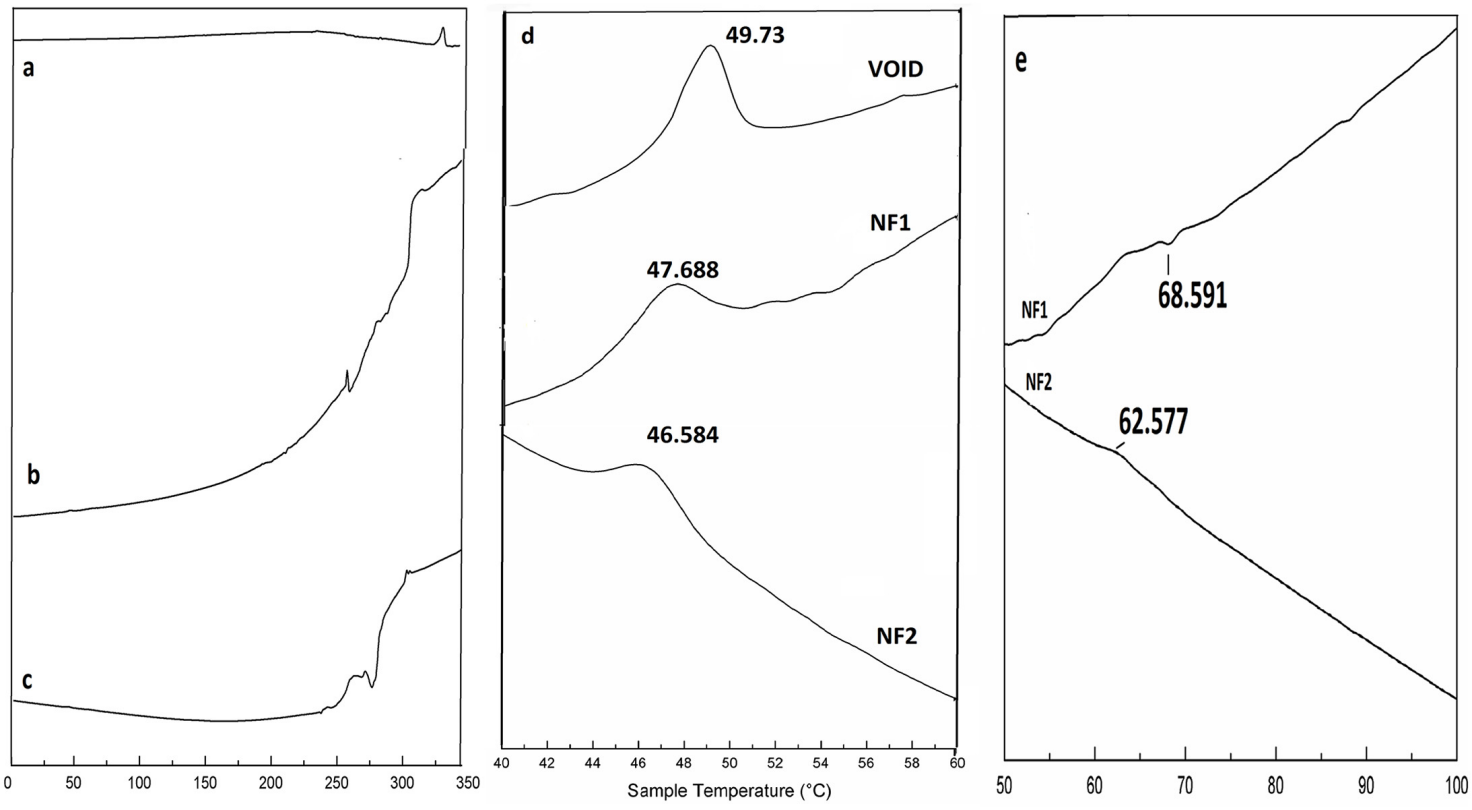
Differential Scanning Calorimetry traces in the region of 0 to 350°C. DSC traces of (a) free Q (b) NF1 and (c) NF2. (d) The trace between is 40 to 60°C is zoomed to highlight PLGA melting temperature in void NPs, NF1 and NF2. (e) The trace between is 50 to 100°C is zoomed to highlight Protein melting temperature for NF1 and NF2.

TGA scans in Fig 7a, 7b and 7c and are representative of melting curves of void NPs, Q and Q-loaded NPs respectively. The TGA spectra of PLGA follows a sharp loss in weight at around 50°C with temperature where as Q shows a slower loss in weight at higher temperature (320°C) in accordance to earlier reports [23, 24]. TGA profile of Q-loaded NPs follows weight loss at intermediate temperature and less steep than PLGA confirming encapsulation of Q. DSC profiles of pure Q in Fig 8a reflect distinct melting temperature of Q at 326°C in agreement with earlier reports [25]. Fig 8b and 8c are representatives of DSC patterns of NF1 and NF2, respectively. The zoomed peaks for PLGA at 50°C and BSA/His between 60–70°C are traced in Fig 8d and 8e respectively as observed by others [26, 27]. The melting peaks for ADR and MTX are merged with that of Q between 300–320°C.

### Drug entrapment efficiency and loading efficiency

Q was efficiently loaded in PLGA NPs, reaching a loading of 105 μg of Q per mg of NP with encapsulation efficiency of 85%. ADR was coated on Q-loaded NP with efficiency of 23.2%. MTX was successfully coated on NPs with efficiency of 84.62%.

### In vitro release kinetics study

**Fig 9:**
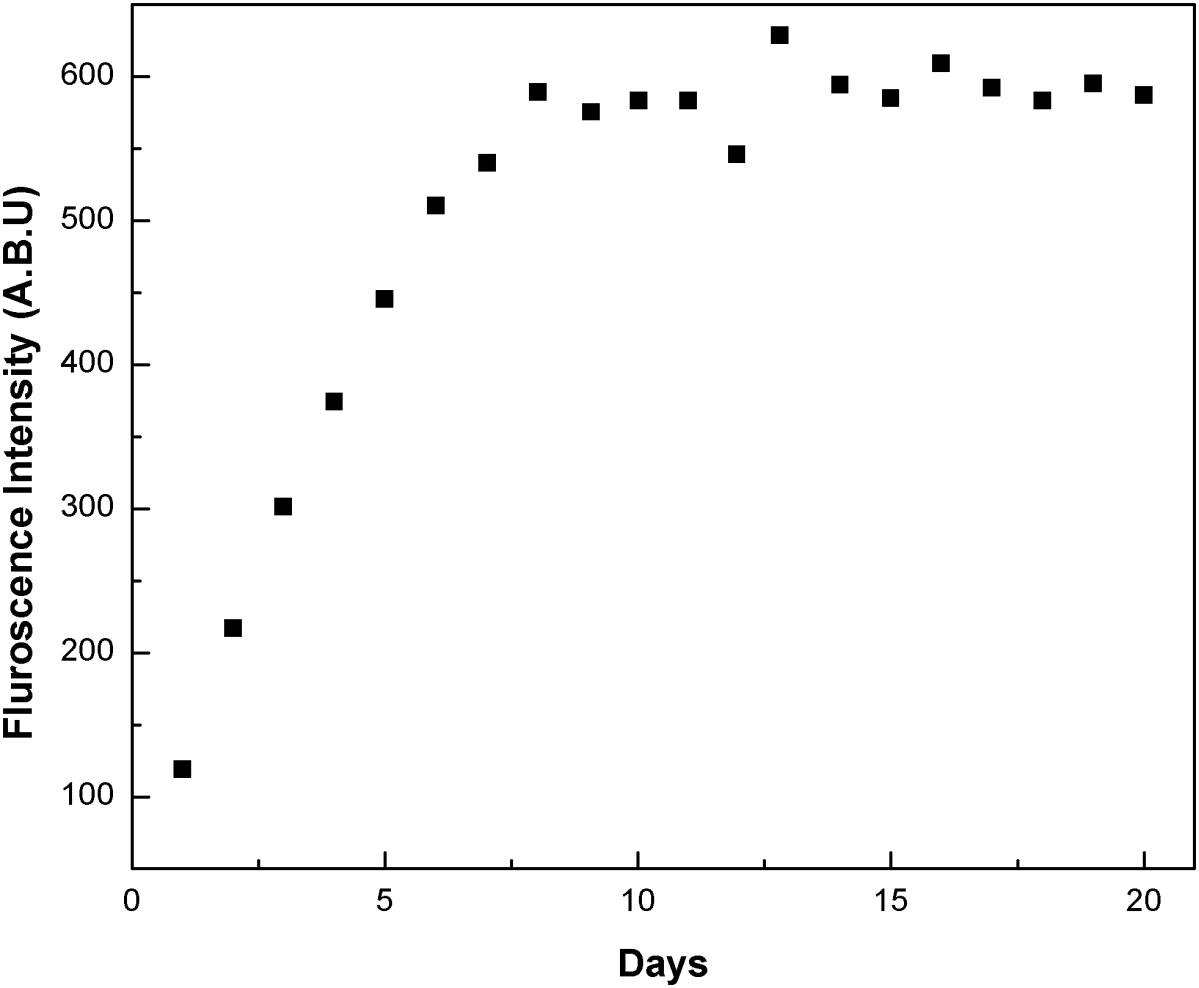
Release of Q as measured fluorimetrically in the supernatant collected on regular intervals for 20 days. 2 mg of Q-loaded NPs were dissolved in 1 ml of PBS (0.01 M, pH 7.4) containing 0.1% v/v of NaN_3_ and kept in shaker incubator and centrifuged before supernatant was collected.

*In vitro* drug release kinetics followed for 20 days is represented in Fig 9 demonstrating initial burst of drug from PLGA NP, followed by gradual release of drug on subsequent days. This is the characteristic feature of PLGA NPs, which can vary with composition of PLGA and cellular environment [28].

## Discussion

Targeted drug delivery has gone through many facets over the last few decades; the latest being nanotechnology. This technology has advantage of formulating tailor made drugs to suit the purpose from the existing conventional anti cancer drugs. The main features of the formulations are size, charge and composition which renders the conventional drugs into potential drugs that can be consumed orally. The nano particles can be of any biodegradable polymer like chitosan, lipids, synthetic PLGA, PEG etc. in which more than one drug can be encapsulated. In the present study Q-loaded PLGA NPs are surface modified to accommodate a second drug on the surface. Formation of Q-loaded polymer (PLGA) nanoparticles by the solvent evaporation method is exhibited by the SEM images in Fig 3a and 3b. The surface of the Q-loaded NPs is slightly uneven compared to smooth surface of void NPs. The size determined by DLS (Fig 5) for void NPs and Q-loaded NPs is about 180 and 250 nm respectively. The size of Q-loaded NPs is greater than void NPs confirming encapsulation. FT-IR, TGA and DSC spectra of Q, void NPs and Q-loaded NPs as illustrated in Figs 6, 7 and 8 respectively exhibit features that are supportive of presence of Q and PLGA in the samples. The FTIR spectral bands corroborate with those reported for Q and PLGA and their shifts in Q-loaded NPs is evidence of encapsulation [18–22]. Q inside the NPs has different physical form than the free Q and the shifts are attributed to this. PLGA physical properties themselves have been shown to depend upon multiple factors, including the initial molecular weight, the ratio of lactide to glycolide, the size of the device, exposure to water (surface shape) and storage temperature. The hydrophobic nature of PLGA influences polymer degradation which in turn tunes the slow release of the drug from inside the core. Release of Q from NPs was monitored fluorimetrically and release up to twenty days after initial burst was recorded (Fig 9). The primary requirement for better therapeutic properties of NFs per se controlled release of Q has been achieved as evidenced by release kinetics [28]. Other factors like the surface charge of the NPs also influence their efficacy in cancer treatment.

Nature of NF’s surface is very crucial for their uptake by the cancerous cells. PLGA-NPs with no surface modification; carrying negative charge can be rapidly opsonised and massively cleared by RES, mainly the liver and spleen. This is a major obstacle for active targeting as the system recognises and removes the NPs from systemic circulation, and hinders effective delivery of the nano drug to cancerous cells. Surface modification of these polymer NPs with hydrophilic biopolymers recognized by RES is the most pragmatic way to control opsonisation and favour targeted drug delivery [29–33].

Binding of drugs like adriamycin, metmorfin, aspirin, norfloxacin and ASN with serum albumins is reported [14, 34–37]. The protein-drug complexes are stable and mainly bind by H-bonding, electrostatic and hydrophobic interactions. In recent years, extensive research has been focused on adriamycin delivery via various natural and synthetic delivery tools as nanoparticles in order to aid the drug solubility, improve the therapeutic process by extending the circulation time and enhance uptake into tumours, through the permeability and retention effect [38–44].

In the present study, surface modification is achieved by adsorption of the protein–drug complex on the surface of the Q-loaded NPs. Surface modification of NPs by BSA is already reported to be stable and retains the ability to bind to drugs [3,15–17]. This modification will shield the NPs from being eliminated by RES. Surface modification of these NPs by coating BSA/His bound to ADR/MTX respectively is captured in the SEM images Fig 3c and 3d. The surface modified NFs are larger in size than Q-encapsulated NPs (between 400 to 500 nm) compared to Q-loaded (250 nm). Also the NPs show less aggregation suggesting acquisition of higher charges upon surface modification leading to higher repulsive forces between them. TEM images (Fig 4) of NFs capture three different layers namely the encapsulated drug, the polymer encapsulation and the surface coating of the NPs.

Zeta potential is the charge that develops between solid surface of NPs and its medium of suspension. The net charge at the NP’s surface affects the ion distribution in the near by region by increasing the concentration of counterions close to the surface. The positive zeta potential of NF1 and NF2 facilitates higher interaction with the negatively charged cancerous cells due to over expression of negatively charged glycol proteins compared to Q-loaded NPs with negative zeta potential. MDR is mainly due to the over expression of the these negatively charged plasma membrane glycoproteins, which are capable of extruding various generally negatively charged xenobiotics including some anticancer drugs.

Doxorubicin is a cytotoxic agent with high growth inhibition values is positively charged and may not be flushed away by the negatively charged cancerous cells but can be impeded by its low solubility. In NF1 where ADR bound to BSA is coated on Q-loaded NPs, its zeta potential is positive (+3.0 mv) in spite of negative charges on BSA and Q-loaded NPs. More positive zeta potential (+8.0 mv) was observed when MTX bound His was coated on Q-loaded NPs. Also 85% of MTX is bound to His compared to 23% of ADR on BSA. High loading of MTX and higher positive charge on NF2, makes it a better nano formulation than NF1. Once the NFs are inside the cell, both the drugs are delivered and the low solubility of Q is also overcome. The extracellular pH of malignant tumors is significantly lower than that of normal tissues under physiological conditions and helps to stabilize positive charge of NFs. These two factors—more positive charges of NFs at the tumor site and more negative charges of tumor cells/vasculature could lead to tumor-specific accumulation of NFs. This method has been successful in accelerating *in vitro* uptake of coumarin to cancer cells, enhanced cytotoxicity of paclitaxel and increased *in vivo* accumulation of coumarin in tumor-bearing tissues [45].

## Conclusion

Quercetin, a flavonoid with anti-cancer properties, is limited by low solubility and bioavailability to be successfully designated as anti-cancer drug. By encapsulating it in polymer nano particles; this limitation can be been overcome. By surface modification of the Q-loaded NPs; the chances of degradation before reaching the target can be restricted. Finally by achieving positive zeta potential; the NFs are electrostatically favorable to interact with the negatively charged cancerous cells resulting in specific uptake and accumulation. Combination Q in nano form with regular chemotherapeutic drugs like ADR and MTX in NF1 and NF2 respectively may overcome MDR—a daunting task in cancer treatment. Here NF2 is a better formulation than NF1 as it carries higher positive charge as well as larger quantities of MTX are loaded on the surface of NPs compared to ADR in NF1. The application of the NFs on cancerous cell line K562 is in progress.
